# Morphogenic Protein RodZ Interacts with Sporulation Specific SpoIIE in *Bacillus subtilis*

**DOI:** 10.1371/journal.pone.0159076

**Published:** 2016-07-14

**Authors:** Katarína Muchová, Zuzana Chromiková, Niels Bradshaw, Anthony J. Wilkinson, Imrich Barák

**Affiliations:** 1 Institute of Molecular Biology, Slovak Academy of Sciences, Bratislava, Slovakia; 2 Department of Molecular and Cellular Biology, Harvard University, Cambridge, Massachusetts, United States of America; 3 Structural Biology Laboratory, Department of Chemistry, University of York, York, United Kingdom; Institut de Génétique et Développement de Rennes, FRANCE

## Abstract

The first landmark in sporulation of *Bacillus subtilis* is the formation of an asymmetric septum followed by selective activation of the transcription factor σ^F^ in the resulting smaller cell. How the morphological transformations that occur during sporulation are coupled to cell-specific activation of transcription is largely unknown. The membrane protein SpoIIE is a constituent of the asymmetric sporulation septum and is a crucial determinant of σ^F^ activation. Here we report that the morphogenic protein, RodZ, which is essential for cell shape determination, is additionally required for asymmetric septum formation and sporulation. In cells depleted of RodZ, formation of asymmetric septa is disturbed and σ^F^ activation is perturbed. During sporulation, we found that SpoIIE recruits RodZ to the asymmetric septum. Moreover, we detected a direct interaction between SpoIIE and RodZ *in vitro* and *in vivo*, indicating that SpoIIE-RodZ may form a complex to coordinate asymmetric septum formation and σ^F^ activation. We propose that RodZ could provide a link between the cell shape machinery and the coordinated morphological and developmental transitions required to form a resistant spore.

## Introduction

Upon starvation, the rod shaped Gram-positive bacterium *Bacillus subtilis* can enter into a differentiation process termed sporulation. Sporulation begins with asymmetric cell division which leads to formation of two compartments of unequal size, a smaller forespore and a larger mother cell. The forespore is subsequently engulfed by the mother cell in a process similar to eukaryotic phagocytosis. Finally, after a thick proteinaceous shell is deposited around the developing spore, the mother cell lyses releasing a mature, resistant spore. The spore can lie dormant indefinitely and germinate when growth conditions improve [1,2]. The first clear morphological event in this process is the formation of the asymmetric septum. At the onset of sporulation FtsZ, the eukaryotic tubulin like homologue, is localized at mid-cell where it forms a ring-like structure termed the “Z-ring” [3]. Z-rings then move as spiral-like structures from mid-cell towards the cell poles where they reassemble as two separate rings near the two poles of the cell [4]. The switch of cell division to the polar sites is under the control of σ^H^ and Spo0A which turn on transcription of *spoIIE* and enhance expression of *ftsZ* from a second sporulation-specific promoter [4,5]. SpoIIE is an 827 residue membrane protein that consists of three domains [6,7]; it has 10 putative membrane-spanning segments (domain I, residues 1–330) at its amino terminus and a PP2C-type phosphatase domain of known structure (domain III, 590–827) at its C-terminus [8]. The central domain II, whose boundaries are not precisely defined, is conserved only among SpoIIE orthologues. Domain II is required for localization of SpoIIE to the divisome and its reported interaction with FtsZ [6,9]. SpoIIE co-localizes with FtsZ [9–11] and moves together with it on a helical trajectory to the polar sites where it forms E-rings that coincide with the Z-rings [4]. Although Z- and E- rings are formed near both cell poles, division occurs only at one cell pole. The maturation of the second polar division site is blocked by one or more σ^E^-dependent gene products [12]. The asymmetric septum can be seen as a thinner version of the vegetative septum in which most of the peptidoglycan is removed soon after septation is complete. SpoIIE is an integral component of the asymmetric septum and *spoIIE* deletion mutants are defective in sporulation and give rise at low frequency to aberrantly thick asymmetric septa reminiscent of vegetative septa [13–15]. SpoIIE remains at the polar septum until septation is complete and later becomes redistributed throughout the forespore membrane as it performs its role in activation of the first compartment-specific sigma factor, σ^F^ [16]. A third possible role for SpoIIE emerges from the discovery that SpoIIE is subsequently recaptured at the forespore face of the polar septum where it may participate in peptidoglycan remodelling [17].

The rod shape of *B*. *subtilis* cells is maintained throughout its life cycle. Thus, factors that control cell shape must be present in all phases of cell growth. As in other bacteria, the cell wall is the principal determinant of *B*. *subtilis* cell shape. The cell wall is composed of a thick peptidoglycan layer to which teichoic acids and cell wall-specific proteins are covalently attached [18]. The coordinated action of two mechanisms of cell wall synthesis, one connected with cell division, the other with cell elongation, is thought to be responsible for maintaining the rod shape [19]. The divisome and the elongasome are large protein complexes responsible for peptidoglycan synthesis, the first acts at the site of division while the second directs insertion of peptidoglycan along the long axis of the cell, thus allowing cylindrical growth [20]. During cell division, the tubulin homologue FtsZ is the main player whereas elongation is driven by the actin homologue MreB and its paralogues MreBH and Mbl [19]. The interaction of MreB with the elongasome consisting of MreC, MreD, RodA, PBP1A, PBP2, RodZ [21–26] and proteins involved in peptidoglycan synthesis such as MurF, MurG and MraY [27,28] is crucial for cell shape determination [29]. However, despite extensive research there is still discussion concerning the dynamics of MreB and its function in cell wall synthesis [30]. In previous work, we characterized the morphogenic protein RodZ from *B*. *subtilis* and demonstrated that RodZ is likely to be an essential protein and an important part of the cell shape determining network in this organism [18].

In this study, we report that perturbation to RodZ expression causes a drop-off in sporulation efficiency. Depletion of RodZ during sporulation resulted in lower frequency formation of asymmetric septa and defective activation of σ^F^. In addition, we show that RodZ localizes to the polar septum during sporulation and co-localizes with SpoIIE. Finally, we demonstrate that RodZ interacts directly with SpoIIE. We propose that as well as being an important cell shape determinant during vegetative growth, RodZ is also required for asymmetric cell division which is a prerequisite for the development of resistant spores.

## Materials and Methods

### Media and general methods

*E*. *coli* strains were grown in LB media [31]. *B*. *subtilis* cells were grown in either Difco sporulation medium (DSM) or Spizizzen´s minimal salts medium (SMM) [32]. Transformation of *B*. *subtilis* and other standard genetic techniques were carried out as described previously [32]. When required, media were supplemented with spectinomycin (100 μg ml^-1^), kanamycin (10 μg ml^-1^), chloramphenicol (5 μg ml^-1^) or erythromycin (1 μg ml^-1^) and lincomycin (25 μg ml^-1^). P_spac_ driven expression was induced using 0.2–1 mM isopropyl β-D-1-thiogalactopyranoside (IPTG). 0.1–0.3% xylose was used for the induction of P_xyl_ expression.

The sporulation efficiency was measured by growing cells in DSM or SMM supplemented with 1mM Ca(NO_3_)_2_, 10 μM MnCl_2_ and 1 μM FeSO_4_ (SMM+salts) for 24h at 37°C. The total number of viable cells (colony forming units) was estimated by plating the cell cultures onto LB plates. The number of heat-resistant colony forming units was obtained after incubation at 80°C for 15 min and subsequent plating onto LB plates.

### Bacterial strains and plasmids

The *B*. *subtilis* and *E*. *coli* strains, plasmids and the sequences of oligonucleotides used in this work are given in Tables A-C in S1 File. The plasmids were constructed using standard methods [31] and amplified in *E*. *coli* MM294 or DH5α cells. All PCR fragments if not mentioned otherwise, were amplified from *B*. *subtilis* chromosomal DNA.

To replace *spoIIE* at the native locus with *spoIIE-ypet*, we took advantage of the recombinant plasmid pXIIE-Dendra prepared for other studies. The final 611 nucleotides of *spoIIE* (omitting the stop codon) were PCR amplified using the primers IIECpxNcoF2 and IIECpxKpnR and ligated into the vector pX-Dendra a-C2 (kind gift from S. Holden), so as to create a fusion of the SpoIIE C-terminal domain to Dendra. To replace Dendra by Ypet, *ypet* was PCR amplified from expression vector pRod49 (kind gift from Mark Leake) using the primers YpetKpnFor2, YpetPstRev2, and ligated into the pXIIE-Dendra. This resulted in the plasmid pXIIE-Ypet, where 611 nucleotides encoding the C-terminus of SpoIIE are in fusion with Ypet. Subsequently, pXIIE-Ypet was digested with EcoRI and XbaI and the resulting 1059 bp fragment containing the 3’ 316 bp of *spoIIE* in an in frame fusion with *ypet* was ligated into EcoRI, XbaI digested pSG1151 [33] to create the integration plasmid pSGIIE-Ypet.

To analyze the interactions of RodZ and SpoIIE in sporulating *B*. *subtilis* cells, *cfp-rodZ* was inserted into BamHI, SacII digested pAX01 [34] by isothermal assembly, and *spoIIE-3XFLAG* including the *spoIIE* promoter (216 nucleotides upstream) was inserted to BamHI, BlpI digested pDG1728 [35]. Constructs were used to transform PY79 and resulting transformants were confirmed to be inserted by double crossover recombination.

To analyze the interactions of cyt-RodZ and cyt-SpoIIE (regions II+III) by pull down methods, pETspoIIE-S and pETrodZspoIIE-S were constructed. A PCR fragment containing the cytosolic part of the *spoIIE* gene was prepared using the primers cytspoIIESX and cytspoIIEEX. To yield pETspoIIE-S and pETrodZspoIIE-S, this PCR fragment was digested with XhoI and cloned into similarly cut pETDuet-1 (Novagen) and pETrodZ [18], respectively.

To express His-tagged cyt-SpoIIE for the determination of the dissociation constant of the cyt-RodZ: cyt-SpoIIE complex by MicroScale Thermophoresis, pETspoIIE was constructed. The PCR fragment containing the cytosolic part of the *spoIIE* gene was prepared using the primers cytspoIIESB and cytspoIIEEB. To yield pETspoIIE, this PCR fragment was digested with BamHI and cloned into similarly cut pETDuet-1 (Novagen).

### Bacterial two hybrid system

Fusions of *B*. *subtilis* RodZ, cyt-RodZ, SpoIIE and domains of SpoIIE to the T25 and T18 fragments of adenylate cyclase were constructed in the BACTH bacterial two hybrid system [36]. PCR fragments obtained by amplification of the genes of interest were cloned into similarly digested pKT25, pKNT25, pUTC18 or pUT18 vectors. To test for protein-protein interactions, transformants of *E*. *coli* BTH101 were plated onto LB plates supplemented with 40 μg ml^-1^ X-Gal (5-bromo-4-chloro-3-indolyl-β-d-galactopyranoside), 0.5 mM IPTG, 100 μg ml^-1^ampicillin and 30 μg ml^-1^ kanamycin and grown for 24–72 hours at 30°C.

### Quantitative β-galactosidase assay

β-galactosidase activity was measured as described previously [37] with an extra wash step added. To eliminate error due to the effects of different carbon sources in the growth medium, the cells were pelleted and resuspended in an assay buffer prior to further processing.

### Western blotting

Western blot analysis was performed essentially as described previously [38]. Briefly, cell cultures were grown in SMM+salts. To deplete RodZ, the cell culture was initially grown with 1 mM IPTG for 2.5 hours and then diluted into a medium lacking IPTG and incubated for an additional 12 hours. The cells were resuspended in 50 mM Tris-HCl pH 8.0, 1 mM EDTA, 200 mM NaCl, 1 mM AEBSF to an A_600_ equivalent to 20 and lysed by sonication. Membrane proteins were solubilized with 1% digitonin. The intracellular levels of Ypet fusion proteins were determined with monoclonal anti-GFP antibody (1:1000) (Roche Diagnostics). The levels of Spo0A were determined using rabbit polyclonal anti-Spo0A antibodies (1:500).

### Protein isolation and purification

*E*. *coli* BL21 (DE3) strains harboring expression plasmids were grown in LB medium at 37°C. When the OD_600_ of the culture reached 0.5, expression of recombinant proteins was induced by the addition of 1 mM IPTG. After 3 hours of further growth at 37°C for cyt-RodZ or overnight growth at 16°C in case of cyt-SpoIIE, the cells were harvested by centrifugation. Cell pellets were resuspended in lysis buffer (20 mM Tris-HCl, pH 8.0, 150 mM NaCl) before being disrupted by sonication. The cell lysate was clarified by centrifugation for 30 min at 80,000 x g. His-tagged proteins were purified using a 1 ml Ni Sepharose HP column (Amersham Biosciences). Proteins were eluted with a 4 ml step-gradient of 40 mM to 1 M imidazole. Co-eluted proteins were identified by Western blot analysis using monoclonal antibodies against the His-tag or the S-tag (Novagen).

For co-immunoprecipitation experiments from sporulating *B*. *subtilis* cells, 1ml of cultures of NB1418 and NB1641 were harvested 2.5 hours following resuspension in SMM. Cells were lysed by bead beating in 200μl of 50 mM Tris-HCl, pH 8.5, 200 mM NaCl, supplemented with complete protease inhibitors (Roche). Membrane proteins were solubilized with 2% digitonin and extracts were incubated with anti-FLAG M2 magnetic beads (Sigma Aldrich) for 1 hour at 4°C. Samples were washed thoroughly in lysis buffer with 0.1% digitonin and eluted with by boiling in SDS buffer. Western blots were probed with monoclonal anti-FLAG (Sigma), and polyclonal antibodies to GFP, σ^A^ and ClpP.

### Microscale Thermophoresis

Microscale Thermophoresis (MST) is a novel method that detects changes in the thermophoretic movement of fluorescently labeled molecules in an aqueous solution in the microscale environment with temperature gradient [39]. In this experiment, the isolated cytosolic part of SpoIIE protein (cyt-SpoIIE) was fluorescently labeled with the Monolith NT.115 protein labeling kit (NanoTemper Technologies, Germany) using red fluorescent dye NT-647 NHS (amine-reactive) according to the manufacturer’s instructions. After removal of labeling reagents by buffer exchange chromatography, cyt-SpoIIE was eluted in 25 mM Tris-HCl, pH 8.0, 150 mM NaCl, 40 mM imidazole, 5% glycerol, 0.05% Tween-20. Binding experiments were carried out on a Monolith NT.115 device, using standard capillaries, provided by NanoTemper Technologies. The concentration of labeled cyt-SpoIIE was 34 μM and was kept constant at this level. Concentration of unlabeled cyt-RodZ, which functioned as a titrant, varied in the range from 6.7 nM to 221 μM. During the MST assay, the led power was shown to be efficient at 95% and laser power at 40%. The experiment was carried out in 16 capillaries, each of which was filled with reaction buffer containing 34 μM cyt-SpoIIE and cyt-RodZ in one of its varying titration concentrations. Fluorescence of labeled cyt-SpoIIE molecules was detected while the IR laser was turned off. After the IR laser was turned on for 30 s, and the region in the capillary was heated, molecules started to move along the temperature gradient away from the heated region to the colder periphery of the capillary and the fluorescence thus decreased in the heated region over a certain period of time. After switching the laser off, molecules returned to the region because of the process of back diffusion. The relative change of fluorescence in each capillary was plotted as the normalized fluorescence, F_norm_, which is the ratio of fluorescence before induction of the temperature gradient and the fluorescence detected during the temperature gradient. The preliminary K_d_ was determined by nonlinear fitting of the thermophoretic responses using the NT Analysis software.

### Fluorescence microscopy and image acquisition

*B*. *subtilis* cultures were grown as liquid cultures in appropriate media as described above. To deplete RodZ, the relevant culture was grown in SMM+salts with 1 mM IPTG for 2.5 hours and then diluted into a medium lacking IPTG and incubated at least for an additional ten hours. For membrane visualization, the fluorescent dye FM 4–64 (Molecular Probes) was used at concentrations of 0.2–1 μg ml^-1^. Cells were examined under the microscope on 1% agarose covered slides. When it was necessary to increase the cell density, cells were concentrated by centrifugation (3 min at 2,300 x g) and resuspended in a small volume of supernatant prior to examination by microscopy. All images were obtained with an Olympus BX63 microscope equipped with a Hamamatsu Orca-R^2^ camera. Olympus CellP imaging software or Olympus Image-Pro Plus 6.0 software was used for image acquisition and analysis. Line scans were completed using the line scan function in Olympus CellP imaging software. Line scan measurements were exported to Microsoft Excel for display.

### Time-lapse microscopy

Time-lapse fluorescence microscopy was carried out with a Zeiss LSM 510 Meta confocal system with a Zeiss Axiovert inverted microscope, fitted with a Plan Apochromat 100x / 1.4 oil objective and a temperature-controlled stage. The cells were prepared as described above. A 488 nm laser was used for excitation of GFP and emission collected through a 495–600 nm bandpass filter. A 514 nm laser was used for excitation of Ypet and emission collected through a 520–600 nm bandpass filter.

## Results

### Depletion of RodZ affects sporulation

RodZ is a membrane protein that consists of three domains; a well-conserved N-terminal cytosolic domain (residues 1–91, cyt-RodZ), a transmembrane domain (92–111) and an extracellular C-terminal domain (112–288). In previous work we have shown that in addition to its role in cell shape maintainance, RodZ influences vegetative cell division [18].

In this work, we asked if RodZ plays any role in asymmetric cell division, which is the first clear morphological feature of endospore development. Thus, we determined the sporulation efficiency of two previously studied strains in which the production of RodZ is perturbed [18], IB1457 expressing a cytosolic fragment of RodZ (*cyt-rodZ*) and a conditional mutant strain IB1458 (*P*_*spac*_*-rodZ*) where expression of the *rodZ* gene is under the control of an IPTG-inducible promoter. Since the effect of *rodZ* mutations is more evident in SMM minimal medium, we cultivated cells in this medium supplemented with 1 mM Ca(NO_3_)_2_, 10 μM MnCl_2_ and 1 μM FeSO_4_ (SMM+salts), salts that are commonly added to DSM sporulation medium. The sporulation efficiency of the *cyt-rodZ* strain (IB1457) in both media, SMM+salts and DSM, was comparable to that of the wild type strain (Table 1). This extends our previous finding that cyt-RodZ is sufficient for viability [18] showing that the truncated protein also supports efficient sporulation.

**Table 1:** Sporulation efficiency of *B*. *subtilis* strains.

Strain	Viable cells^a^ (cfu/ml)	Spores^a^ (cfu/ml)	Sporulation^a^ (%)
SMM+salts medium			
wild type	(2.4 ± 0.5) x 10^8^	(7 ± 2.1) x 10^7^	28 ± 2.8
*cyt-rodZ*	(1.4 ± 0.6) x 10^8^	(3.8 ± 1.5) x 10^7^	27 ± 5.5
*P*_*spac-*_*rodZ* + IPTG T_-2.5_	(1.9 ± 0.8) x 10^8^	(2.5 ± 0.8) x 10^7^	13.7 ± 3.1
*P*_*spac-*_*rodZ—*IPTG T_-2.5_	(2.4 ± 1.4) x 10^8^	(2.2 ± 0.4) x 10^6^	0.9 ± 0.5
*P*_*spac-*_*rodZ* + IPTG T_0_	(1.7 ± 0.4) x10^8^	(2.9 ± 0.4) x 10^7^	18.3 ± 5.7
*P*_*spac-*_*rodZ*—IPTG T_0_	(1.8 ± 0.8) x 10^8^	(0.7 ± 0.5) x 10^6^	0.3 ± 0.2
DSM medium			
wild type	(4.7 ± 1.9) x 10^8^	(4 ± 1.3) x 10^8^	88 ± 7
*cyt-rodZ*	(3.2 ± 0.5) x 10^8^	(2.6 ± 0.4) x 10^8^	83 ± 14
*P*_*spac-*_*rodZ* + IPTG T_-2.5_	(3.4 ± 0.2) x 10^8^	(2.4 ± 0.3) x 10^8^	72 ± 12
*P*_*spac-*_*rodZ—*IPTG T_-2.5_	(8.7 ± 0.9) x 10^7^	(2.6 ± 1.2) x 10^7^	30.5 ± 14
*P*_*spac-*_*rodZ* + IPTG T_0_	(4.8 ± 0.6) x 10^8^	(3.5 ± 0.7) x 10^8^	70 ± 2.5
*P*_*spac-*_*rodZ*—IPTG T_0_	(1.8 ± 1.5) x 10^8^	(4.4 ± 3.7) x 10^7^	24 ± 4.5

Cells were depleted of RodZ at the times indicated T_-2.5_ (2.5 h of growth) and T_0_ of sporulation. Sporulation efficiency was determined after 24 hours.

^a^ Values represent the average ± standard deviation (SD) of three independent experiments

To measure the sporulation efficiency of cells depleted of RodZ, cell cultures of the *P*_*spac*_*-rodZ* strain (IB1458) were initially grown in SMM+salts or DSM containing 1 mM IPTG. After 2.5 hours of growth or after reaching the stationary phase, cells were diluted into corresponding media lacking IPTG and grown for an additional 24 hours. We observed that the final OD_600_ of culture depleted of RodZ (OD_600_ 1.4) was similar to that of wild type and culture grown in the presence of IPTG, respectively (OD_600_ 1.6 respectively1.5). Although the total number of viable cells was comparable under all conditions tested, a decrease in the sporulation efficiency was observed (Table 1). The determined sporulation defect was similar under two conditions of RodZ depletion; when the cell culture was depleted of RodZ after 2.5 hours of growth as well as if depletion was delayed until the culture entered into stationary phase. The strongest sporulation defect was observed when the culture was grown in the absence of IPTG in SMM+salts with sporulation reduced to 0.3–0.9% while in DSM this defect was more moderate and sporulation was lowered to 24–30% (Table 1). Growth in the presence of 1 mM IPTG restored the sporulation efficiency to 13–18% and 70–72% in SMM+salts and in DSM, respectively. The higher sporulation efficiencies observed for the *P*_*spac*_*-rodZ* strain (IB1458) grown in DSM are probably due to leakiness of the P_spac_ promoter in this medium as has been reported previously [40,41]. The drop in the sporulation efficiency of this strain in SMM+salts indicates that lowering the concentration of RodZ disturbs sporulation. Taken together, our experiments indicate that RodZ is important for efficient sporulation. However, as we have been unsuccessful in constructing a *rodZ* deletion mutant strain [18], we cannot fully evaluate the contribution of RodZ to sporulation.

### RodZ is required for the compartment-specific activation of σ^F^

**Fig 1:**
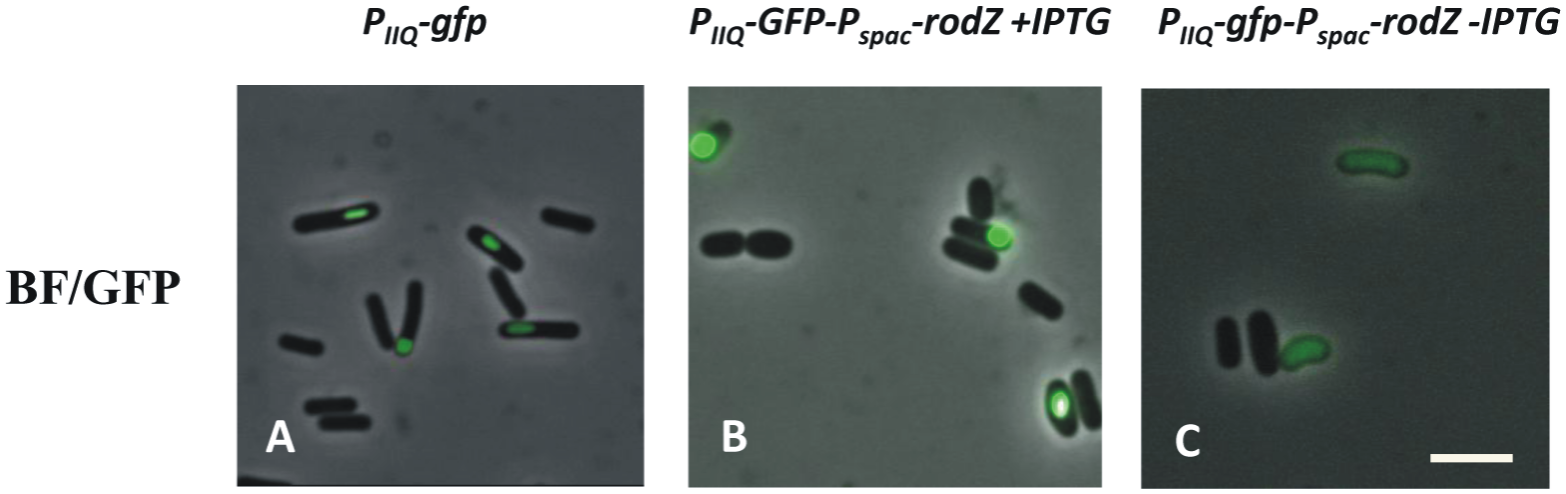
RodZ is required for compartment specific activation of σ^F^. Compartment specificity of σ^F^ activation was monitored in cells producing GFP under the control of the forespore-specific P_spoIIQ_ promoter (P_IIQ_) in wild type *B*. *subtilis* IB827 (A) and in strain IB1563 (B and C), in which expression of the *rodZ* gene is under the control of an IPTG-inducible promoter, grown in SMM+salts in the presence (B) and absence (C) of 1 mM IPTG. In wild type cells (A) and IB1563 cells grown in the presence of 1 mM IPTG (B) GFP is produced exclusively in the forespore. Under conditions of RodZ depletion, no GFP signal was observed in the majority of cells. For those cells exhibiting green fluorescence, it is distributed throughout the cell indicating uncompartmentalized activation of σ^F^ (C). The scale bar represents 4 μm.

The first clear morphological event in sporulation is the formation of the asymmetric sporulation septum which represents the stage II of sporulation [1]. Upon completion of the asymmetric septum, σ^F^ is activated in the forespore. Although σ^F^ becomes active only in the forespore, it is present in the pre-divisional cell and partitions into both compartments following the asymmetric septation. To investigate the possible role of RodZ in asymmetric septation and σ^F^ activation, we prepared strain IB1563 in which *gfp* is under the control of a σ^F^-controlled promoter (P_spoIIQ_) and expression of *rodZ* is under the control of an IPTG-inducible promoter. We monitored forespore-specific production of GFP in the cells when RodZ was depleted. Cell cultures were grown in SMM+salts containing 1 mM IPTG. After 2.5 hours of growth, cells were diluted into medium lacking IPTG and grown for an additional 14 hours. In a control wild type strain (IB827), 95% of cells that produced GFP (113 from a total of 119 cells examined) did so exclusively in the forespore (Fig 1A). In the strain harboring *P*_*spac*_*-rodZ P*_*spoIIQ*_*-gfp* (IB1563) grown in the presence of IPTG, of 180 cells examined, 53 produced GFP, and 88% (47) of these cells produced it exclusively in the forespore (Fig 1B). In addition to this mild effect on compartmentalized σ^F^ activation, these cells were wider and rounder than wild type cells which might be caused by differences in RodZ levels in these two strains. Under conditions of RodZ depletion, in 85% of cells (180 of 211 cells) no GFP signal was observed. GFP was produced in 31 out of 211 cells and the signal was distributed throughout the cell in 90% of these cells (28 cells) indicating uncompartmentalized activation of σ^F^ (Fig 1C). Staining of the cells with the membrane dye FM4-64 revealed that under conditions of RodZ depletion, asymmetric septa, engulfed forespores or spores were present only in 0.5% (n = 123) of the cells compared to 20% (n = 180) in the control strain (IB827). However, when these cells were cultivated for longer times (total 24 hours), 20 cells with asymmetric septa or engulfed forespores and 6 spores (n = 420) were observed consistent with the measured sporulation efficiency (Table 1). This implies that upon removal of IPTG and depletion of the protein, sufficient RodZ persists in a proportion of cells to support delayed asymmetric septation leading to spore formation.

### RodZ localizes at the asymmetric septum

**Fig 2:**
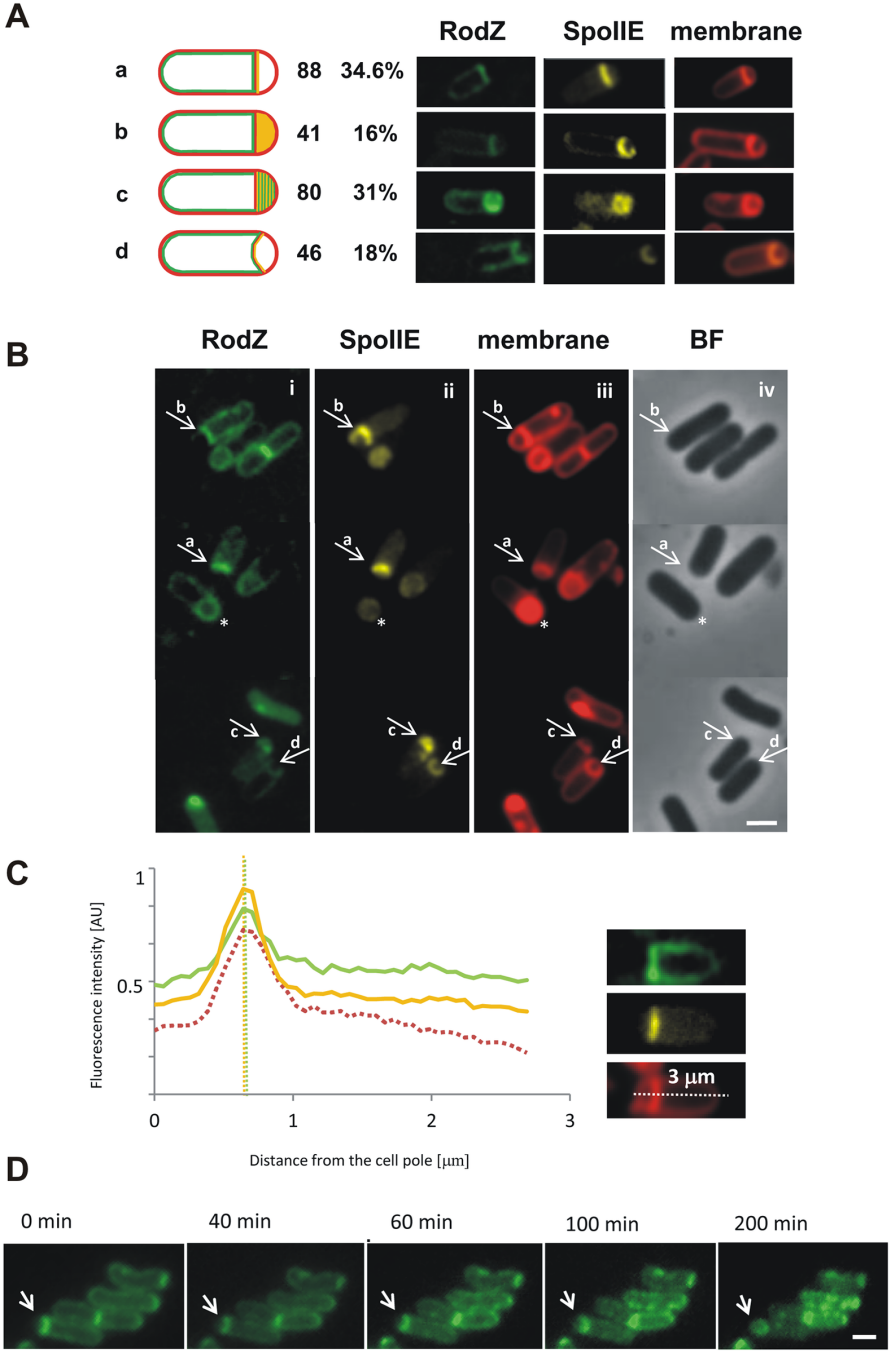
Localization of RodZ and SpoIIE in sporulating cells. A) Differential localization of SpoIIE-Ypet and CFP-RodZ in cells (IB1538) in stage II of sporulation. Cells were harvested 2 hours after the onset of stationary phase. The localization of fluorescence signals in 256 cells in stage II of sporulation was examined and each cell was assigned to one of four classes (a-d) according to the fluorescence distribution. a) cells in which CFP-RodZ is in the mother cell membrane and both SpoIIE-Ypet and CFP-RodZ are at the polar septum; b) cells in which SpoIIE-Ypet is distributed throughout the forespore membrane and CFP-RodZ is confined to the septum and mother cell membrane; c) cells in which SpoIIE-Ypet and CFP-RodZ are distributed throughout the forespore membrane; d) cells in which SpoIIE-Ypet and CFP-RodZ are re-captured to the now curved polar septum. Fluorescence images for representative cells from each class are shown in the panels to the right (left to right): CFP-RodZ (CFP-RodZ signal has been false-colored green); SpoIIE-Ypet; membranes visualized using FM4-64 dye. The cells belonging to each class were counted and expressed as a percentage of the total cell count. B) CFP-RodZ and SpoIIE-Ypet localization. In panels i-iv the same field has been visualized using different fluorescence signals and bright field phase contrast. This field was chosen to illustrate the breadth of phenotypes. Panel i: CFP fluorescence (CFP-RodZ signal has been false-colored green), panel ii: Ypet fluorescence, panel iii: membranes visualized using FM4-64, panel iv: bright field phase contrast image. The cells were classified according to the patterns of CFP-RodZ and SpoIIE-Ypet localization as diagrammed in A with representative cells of the different classes indicated by arrows and lower case letters. A cell with an engulfed forespore (stage III) is marked with asterisk. The scale bar represents 1 μm. C) Line scan analyses of normalized fluorescence intensity along the cell axis defined by the white dashed line shown in the lower panel on the right. The fluorescence signal for CFP-RodZ was accumulated at the septum and was more than twice that in the cell membrane. Analysis was performed for 12 cells. The fluorescence intensity was measured along the entire length of the bacterium in three channels: SpoIIE-Ypet (yellow line), CFP-RodZ (green line) and the FM4-64 membrane stain (dotted red line). Right panels show fluorescence images of the cell in which an asymmetric septum has formed and signals for SpoIIE-Ypet, CFP-RodZ and the membrane stain are overlapping: CFP-RodZ (false-colored green), SpoIIE-Ypet and FM4-64. D) Localization of GFP-RodZ in sporulating cells. Cells (IB1450) were harvested 2 hours after the onset of stationary phase and mounted on 1% agarose covered slides for monitoring by time-lapse microscopy. Fluorescence images were acquired at the times indicated, with time 0 representing the beginning of the time lapse experiment. The dynamic localization of GFP-RodZ as it develops from a cell belonging to class a) to the engulfed forespore (the cell is indicated by arrow). The scale bar represents 1 μm.

To examine RodZ localization in sporulating cells, we analyzed GFP-RodZ or CFP-RodZ in cell cultures grown in DSM sporulation medium. GFP-RodZ was produced under the control of a xylose inducible promoter at the ectopic, non-essential *amyE* locus [18]. CFP-RodZ was produced under the control of a xylose inducible promoter at the ectopic *lacA* locus. The sporulation efficiency of strains producing GFP-RodZ or CFP-RodZ was comparable to that of the wild type strain (73% and 77% of the wild type, respectively). For GFP-RodZ or CFP-RodZ, we observed a dynamic localization pattern. In vegetatively growing cells, GFP-RodZ localizes in helical patches within the membrane, at the mid-cell site of septation and at the cell poles [18]. As sporulation proceeds, it moves from the cell pole to possible asymmetric division sites. Later accumulation of the fusion protein at the straight and subsequently the slightly curved, polar septum can clearly be observed with the fluorescence signal at the septum estimated to be more than twice that in the cell membrane (Fig 2A and 2B; cells marked a and b; Fig 2C). However, GFP-RodZ (CFP-RodZ) signal is also seen in the mother cell membrane indicating RodZ’s continuing role in cell shape maintenance (Fig 2). Finally, GFP-RodZ (CFP-RodZ) localizes along the engulfing membrane and fluorescence signals can be seen around the forespore (Fig 2B, panel i cells marked c, d and cell marked *; time lapse Fig 2D).

### CFP-RodZ co-localizes with SpoIIE-Ypet

The pattern of RodZ localization in sporulating cells can be compared to that of SpoIIE. To study the possible co-localization of SpoIIE and RodZ, we prepared strain IB1538 in which SpoIIE is fused to Ypet, a photostable derivative of YFP [42], produced under the control of its native promoter and CFP-RodZ is produced under the control of a xylose inducible promoter at the ectopic *lacA* locus. The sporulation efficiency of this strain was measured and found to be comparable to that of the wild type strain (87% of the wild type). SpoIIE-Ypet and CFP-RodZ exhibit a similar pattern of localization (Fig 2B, panels i-iii). As sporulation commences, CFP-RodZ localizes at polar division sites where E-rings are formed. Later CFP-RodZ accumulates together with SpoIIE-Ypet at the polar septum (Fig 2B, panels i-iii, arrow a). Subsequently, prior to the initiation of engulfment (flat polar septa), SpoIIE-Ypet is released from the septum, and distributed throughout the forespore membranes (Fig 2B, panels ii and iii, arrow b) as has been observed previously [17]. We found that CFP-RodZ is similarly released from the septum, but this release is probably delayed as we were able to capture cells in which SpoIIE was released throughout the forespore membrane while RodZ remained at the septal site (around 16% of cells in stage II, n = 256) (Fig 2A and 2B, panels i and ii, arrow b). In contrast, we observed only one cell among 256 cells in stage II of sporulation in which RodZ was released throughout the forespore membrane while SpoIIE remained at the septal site. Further, we observed signals for released SpoIIE-Ypet and released CFP-RodZ in around 31% of cells in stage II (n = 256) (Fig 2A and 2B, panels i- iii, arrow c). This release of both proteins is transient, because as soon as engulfment begins, SpoIIE-Ypet and CFP-RodZ are re-localized to the septal membranes in around 18% of cells in stage II (n = 256) (Fig 2A and 2B, panels i-iii, arrow d). As engulfment is completed, and cells reach what is characterized as stage III of sporulation, weak SpoIIE-Ypet and CFP-RodZ signals can be observed around the forespore (Fig 2B, panels i-iii, cell marked *).

### RodZ localization during sporulation is dependent on SpoIIE

**Fig 3:**
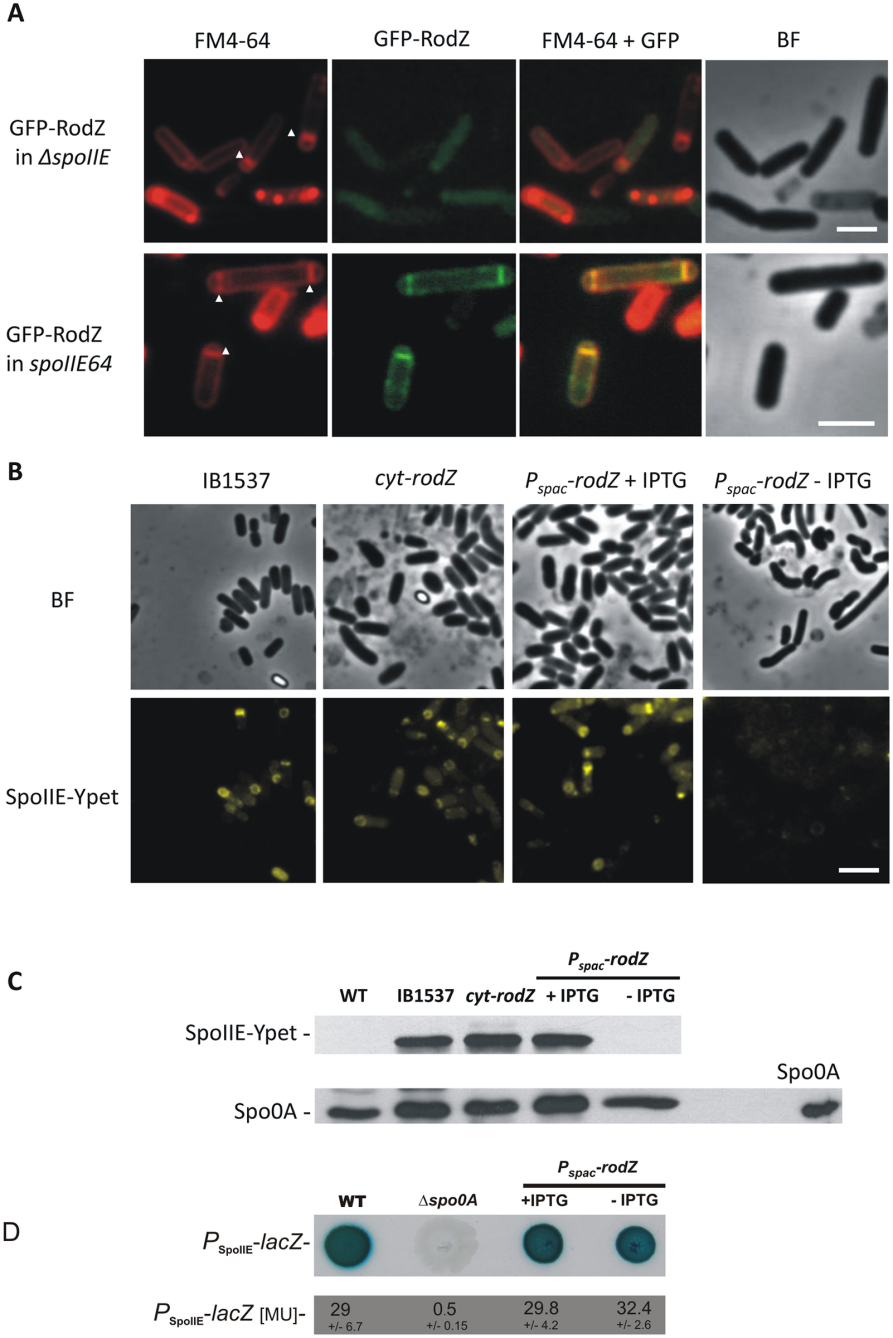
Localization of GFP-RodZ and SpoIIE-Ypet in different mutant strains. A) Cell cultures of strains IB1565 *(ΔspoIIE*, *gfp-rodZ*) and IB1566 (*spoIIE64*, *gfp-rodZ*) were examined 2 hours after the onset of stationary phase. Membranes were stained with FM4-64 (red). Panels in the upper row examine the localization of GFP-RodZ in the *ΔspoIIE* background, revealing that GFP-RodZ is dispersed within the cell. Panels in the lower row examine the localization of GFP-RodZ in the *spoIIE64* background, revealing that GFP-RodZ is localized at the polar septa. Panel FM4-64: membranes visualized using FM4-64, panel GFP-RodZ: GFP fluorescence, panel FM4-64+GFP: overlay of membrane and GFP fluorescence, panel BF: bright field phase contrast. Arrowheads show the position of asymmetric septa. The scale bar represents 2 μm. B) Cell cultures of IB1537 and IB1567 (*cyt-rodZ*) were grown in SMM+salts for 2.5 hours, before dilution into the same medium and growth for an additional 12 hours. Strain IB1568 (*P*_*spac*_*-rodZ*) was initially grown for 2.5 hours in SMM+salts containing 1 mM IPTG, before dilution into medium lacking IPTG and growth for an additional 12 hours. Panel IB1537 shows localization of SpoIIE-Ypet in a wild type background; panel *cyt-rodZ* shows localization of SpoIIE-Ypet in IB1567 expressing the cytosolic part of RodZ; panel *P*_*spac*_*-rodZ* + IPTG shows localization of SpoIIE-Ypet in IB1568 grown in the presence of 1 mM IPTG and panel *P*_*spac*_*-rodZ–*IPTG shows localization of SpoIIE-Ypet in IB1568 grown in the absence of IPTG. In a strain expressing the cytosolic part of RodZ, SpoIIE-Ypet localizes as it does in a wild type background. In cells depleted of RodZ, almost no SpoIIE-Ypet signal was detected. Panels in the upper row: bright field phase contrast; panels in the lower row: Ypet fluorescence. The scale bar represents 4 μm. C) Western blot analysis of SpoIIE-Ypet and Spo0A in PY79 (WT), IB 1537, *cyt-rodZ* and *P*_*spac*_*-rodZ* strains. An anti-GFP antibody was used for detection of SpoIIE-Ypet and an anti-Spo0A antibody was used for detection of Spo0A. Each lane contains the same total protein amount. The control lane on the right was loaded with 0.05μg of purified Spo0A. D) Expression from *spoIIE* promoter. Cultures of cells harboring *P*_*spoIIE*_*-lacZ* fusions in PY79 (WT), *Δspo0A* (IB1599) and *P*_*spac*_*-rodZ* (IB1600) were grown as described above. Cells were spotted onto SMM + salts plates containing X-Gal. β-galactosidase activity was measured as described in Materials and Methods. Numbers indicate Miller units of activity and represent the mean ± SD of activity from three measurements.

Intensive studies of SpoIIE function have led to the identification of a wide repertoire of *spoIIE* mutant strains [13,14,43]. *spoIIE* null mutant strains have a noticeably reduced frequency of septum formation (50 to 70% aseptate cells) indicating that SpoIIE is required for high efficiency initiation of septum formation [13,14]. We analyzed the localization of GFP-RodZ in the *spoIIE* null mutant strain IB1565 (*spoIIE*::Tn*917*Ω*HU7*) in which GFP-RodZ is produced from a xylose inducible promoter. Under sporulation conditions, we observed that in this background GFP-RodZ is dispersed throughout the cell (Fig 3A) and no fluorescence signal was observed in sporulation septa. Next we analyzed the localization of GFP-RodZ in the *spoIIE64* phosphatase activity deficient strain, which contains a single missense substitution that alters residue Leu646 to Phe in the C-terminal phosphatase domain of SpoIIE [44]. This mutant is abortively disporic with the majority of cells assembling thin septa at one or both polar positions [14]. In this background (strain IB1566) GFP-RodZ localizes at both sites of possible asymmetric division (Fig 3A). These results indicate that RodZ localization at the asymmetric septum is dependent on the presence of SpoIIE, but not its phosphatase activity.

### SpoIIE localization in *rodZ* mutant strains

Next we analyzed how SpoIIE localizes in cells with pertubed production of RodZ. Since the effect of *rodZ* mutations is more evident in minimal medium, we used SMM+salts for these localization studies. For this purpose, we prepared strain IB1567 expressing the cytosolic part of RodZ (*cyt-rodZ)* and SpoIIE-YPet under the control of its native (*P*_*spoIIE*_) promoter. In the wider and rounder *cyt-rodZ* cells, SpoIIE assembly at asymmetric septa was maintained (Fig 3B) and we conclude that the cytosolic part of RodZ is sufficient for proper localization of SpoIIE even though the rod cell shape is not fully preserved. We also prepared strain IB1568 expressing *spoIIE-ypet* under the control of its native promoter and *rodZ* under the control of an IPTG inducible promoter. In cells growing in the presence of IPTG, SpoIIE-Ypet localizes as in wild type cells (Fig 3B), however, when RodZ was depleted by the absence of IPTG so that asymmetric septa are formed in only a few cells, almost no SpoIIE-Ypet signal was detected (Fig 3B). Corresponding Western blot analysis revealed that the SpoIIE-Ypet signal in the strain depleted of RodZ is lost (Fig 3C).

To analyze whether RodZ might be influencing the expression of *spoIIE* indirectly by disrupting Spo0A activation, we performed a Western blot analysis using an anti-Spo0A antibody. As shown in Fig 3C, the level of Spo0A in all strains examined was comparable with the level of Spo0A in wild type sporulating control cells. To further verify that RodZ is important for stability of SpoIIE, we inferred the expression levels from *P*_*spoIIE*_ under conditions of RodZ depletion by measuring β-galactosidase activity in strains harboring *P*_*spoIIE*_*-lacZ* reporter fusions (Fig 3D). We found that the levels of β-galactosidase activity were identical in wild type and RodZ-depleted cells. This implies that transcription from *PspoIIE* is not affected by RodZ depletion. From this striking result, we conclude that the loss of SpoIIE-YPet fluorescence signal in Fig 3B and protein staining on the gel in Fig 3C is the result of degradation and/or low stability of SpoIIE in the absence of RodZ in sporulating cells. In summary, we propose that cyt-RodZ or normal levels of RodZ are required for stabilization of SpoIIE.

### RodZ interacts with SpoIIE

**Fig 4:**
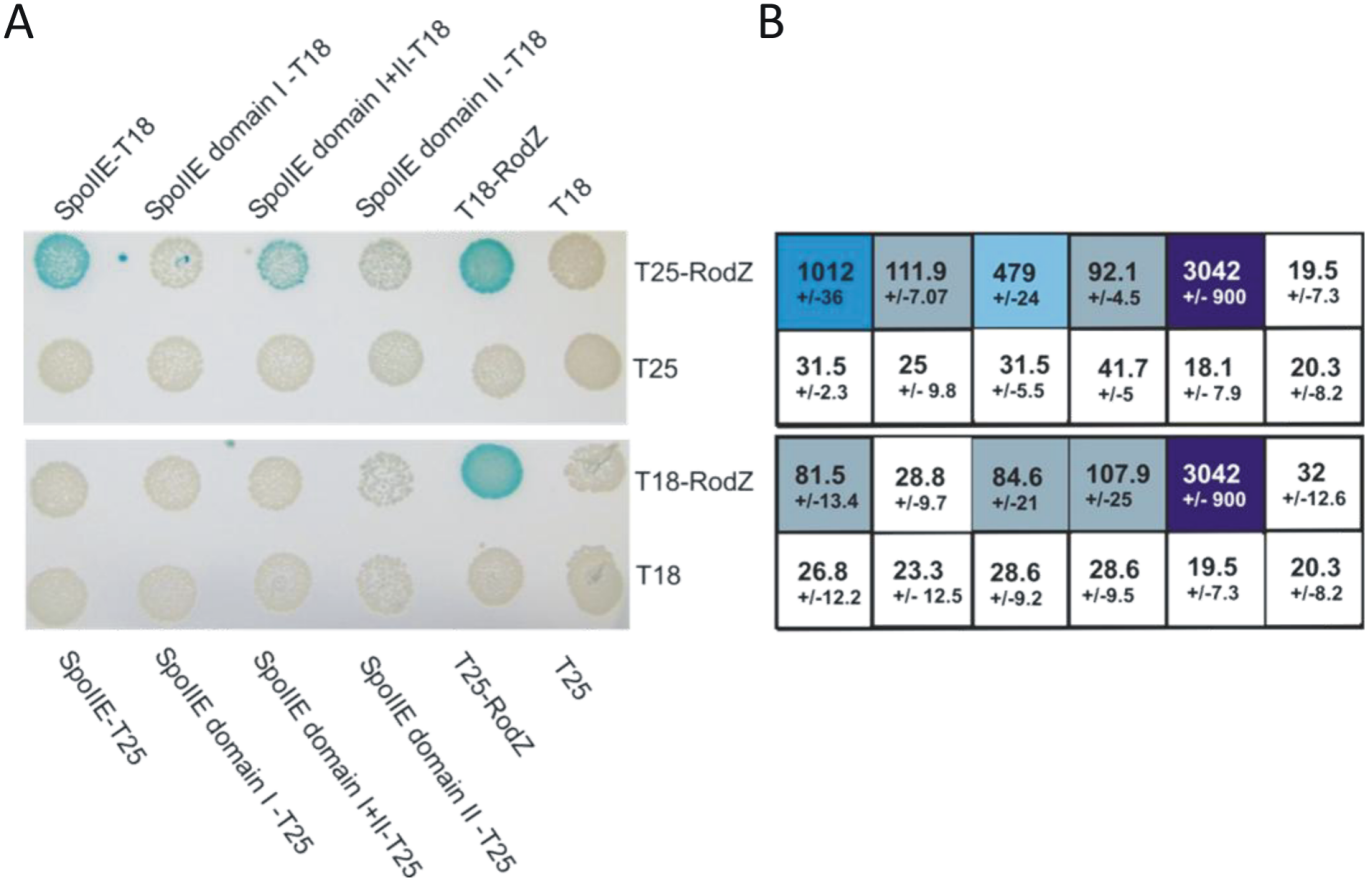
Bacterial two-hybrid analysis of RodZ—SpoIIE protein—protein interactions. *E*. *coli* strain BTH101 *(Δcya*) was co-transformed with plasmids encoding the indicated fusions to adenylate cyclase fragments T18 and T25. A) Colonies were spotted onto selective plates containing IPTG and X-Gal. Blue color indicates a positive interaction between each pair of fusion proteins. B) The interaction strength was quantified using a β-galactosidase assay. Numbers indicate Miller units of activity and represent the mean ± SD of activity from three measurements. Positive interactions are indicated by the intensity of the blue color. The β-galactosidase activity values in the Table correspond to interactions shown in panel A.

To determine whether *B*. *subtilis* RodZ interacts directly with SpoIIE, we employed a bacterial two hybrid analysis (BATCH). Genes encoding RodZ and SpoIIE were cloned in fusion with the T18 and T25 domains of adenylate cyclase, and all combinations of interacting pairs were tested in assays repeated at least three times (see Materials and Methods). These experiments revealed significant interactions between RodZ and SpoIIE (Fig 4). To address the character of their contact in more detail, regions encoding SpoIIE domain I (1–330 aa), domain II (331–608 aa) and domains I+II (1-608aa), were cloned in fusion with both domains of adenylate cyclase, and tested for BATCH interactions in all possible combinations with RodZ and cyt-RodZ. We detected a strong interaction between RodZ and SpoIIE domains I+II, and weaker interactions between RodZ and SpoIIE domains I or II when cloned separately. We also detected interaction between cyt-RodZ and SpoIIE domain II+III, however this was very weak. A positive control experiment is represented by strong RodZ-RodZ interaction (Fig 4).

**Fig 5:**
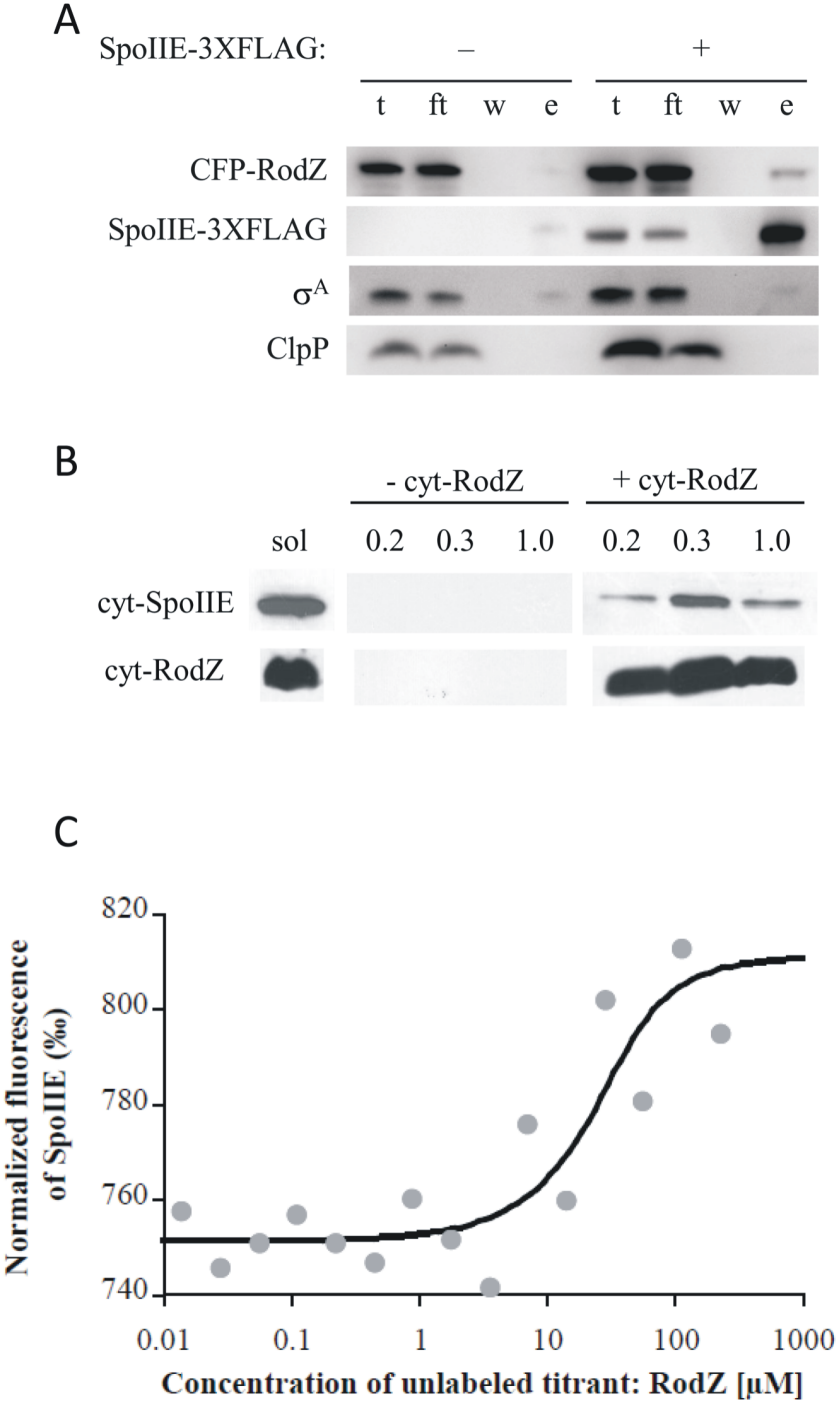
Pull down assay and Microscale Thermophoresis analysis of RodZ-SpoIIE interaction. A) Pull down assay of SpoIIE and RodZ from sporulating cells. Extracts from sporulating cells expressing *cfp-rodZ*, with (right) or without (left) *spoIIE-3XFLAG* were immunoprecipitated with anti-FLAG M2 magnetic beads. Western blots detecting CFP-RodZ and SpoIIE-3XFLAG are shown for total (t), flowthrough (ft), wash (w) and elution (e) fractions. Elution fractions are 20X concentrated relative to the other fractions. σ^A^ and ClpP served as unrelated protein controls. B) Pull down assay of proteins isolated from *E*. *coli* BL21 (DE3). cyt-SpoIIE was S-tagged while cyt-RodZ possessed a His-tag. The pull down assay was performed on a Ni Sepharose HP column. Eluted proteins were probed with an anti-S-tag monoclonal antibody (upper row) and an anti-His-tag (lower row) monoclonal antibody by Western blot analysis. Both proteins were present in the solubilized extracts (sol). S-tagged cyt-SpoIIE is not detected in elution fractions (marked with the concentrations of imidazole 0.2, 0.3 and 1 M) when produced alone (- cyt-RodZ) but is pulled down with His-tagged cyt-RodZ (+ cyt-RodZ) when co-expressed. C) Microscale Thermophoresis. The relative change of fluorescence in each capillary was plotted as the normalized fluorescence, F_norm_, which is the ratio in ‰ of fluorescence before induction of the temperature gradient and the fluorescence detected in the presence of the temperature gradient. If cyt-RodZ interacts with cyt-SpoIIE, F_norm_ changes due to formation of complexes between the fluorescently labeled analyte (cyt-SpoIIE) and unlabeled titrant (cyt-RodZ) creating a binding curve, where F_norm_ correlates with the increasing concentration of titrant. Thus, the signal obtained during the experiment corresponds directly to the fraction of fluorescently labeled molecules of cyt-SpoIIE in complex with the titrant, cyt-RodZ. The binding curve was fitted to the function [AT] = 1/2*(([A0]+[T0]+K_d_)-(([A0]+[T0]+K_d_)2-4*[A0]*[T0])1/2), yielding a value of K_d_ = 10 μM ± 2 μM), where [AT] represents concentration of complexes formed between fluorescent molecules of the analyte [A] and non-fluorescent molecules of the titrant [T]. [A0] is the known concentration of fluorescent molecule and is kept constant during the experiment. [T0] is the known concentration of the titrant which varies in the capillaries. K_d_ is the dissociation constant.

To further examine the interaction of RodZ with SpoIIE, we performed co-immunoprecipitation assays of SpoIIE and RodZ from extracts of sporulating cells. Cells harboring CFP-RodZ and full length FLAG tagged SpoIIE were induced to sporulate by resuspension. Immunoprecipitation of SpoIIE-FLAG from detergent solubilized extracts of these cells also pulled down CFP-RodZ, but not the control proteins σ^A^ or ClpP (Fig 5A), demonstrating an interaction between RodZ and SpoIIE during sporulation. Furthermore, we used the pETDuet system to co-express cyt-RodZ harboring a His_6_-tag with the whole cytosolic part of SpoIIE (cyt-SpoIIE) fused to an S-tag in *E*. *coli*. SDS-PAGE confirmed that both cyt-RodZ and cyt-SpoIIE were produced in *E*. *coli*, either alone or when co-expressed. cyt-RodZ was affinity purified on an immobilised Ni^2+^ column and the protein fractions were analyzed by Western blotting using anti-His-tag and anti-S-tag monoclonal antibodies to detect RodZ and SpoIIE fusions, respectively. The results obtained are shown in Fig 5B and demonstrate that S-tagged cyt-SpoIIE is not detected in elution fractions when produced alone but is pulled down with His-tagged cyt-RodZ when co-expressed (Fig 5B). This result suggests that the cytosolic component of *B*. *subtilis* RodZ directly associates with cytosolic part of SpoIIE.

Formation of a cyt-RodZ—cyt-SpoIIE complex was further analyzed by Microscale Thermophoresis (Fig 5C). In this analysis, His-tagged cyt-SpoIIE (see Materials and Methods) was fluorescently labeled and titrated with successive additions of cyt-RodZ. The resulting thermophoresis signals were plotted against the cyt-RodZ concentration. Increasing concentrations of cyt-RodZ triggered changes in the thermophoretic movement of fluorescently labeled molecules, indicating that more molecules of cyt-SpoIIE were engaged in a complex with cyt-RodZ, which resulted in altered thermophoretic movement of fluorescently labeled cyt-SpoIIE. From the binding curve we determined a value of K_d_ = 10 ± 2 μM (see Materials and Methods). Together these experiments provide multiple independent lines of evidence for a direct interaction between SpoIIE and RodZ, and that this interaction occurs during sporulation.

## Discussion

The overall rod cell shape of *B*. *subtilis* is maintained during vegetative growth as well as during sporulation [45]. Since the cell wall is the main determinant of bacterial cell shape, factors involved in the synthesis and degradation of its major structures play crucial roles in cell shape determination. Peptidoglycan, the essential component of the cell wall, is synthesized during cell division and elongation by large protein complexes, known as the divisome and elongasome respectively, which share more similarities than was previously thought [20]. However, despite extensive research, there are several proteins known to be involved in cell shape maintenance, whose exact functions have not yet been elucidated [30]. Among these is the highly conserved cytoskeletal protein RodZ [46–48]. In *E*. *coli*, the absence of RodZ leads to the formation of round or misshapen cells, while RodZ overproduction results in elongation of the cell [46,47]. A strong cell shape effect has also been observed in *C*. *crescentus*, where RodZ is essential for viability [48]. The membrane topology of RodZ and the direct interaction of its cytoplasmic domain with MreB, a key player in bacterial morphogenesis, suggest that RodZ could be an additional transmembrane stabilizing factor of the bacterial cell wall elongation complex [26,49]. Recently, it was demonstrated that RodZ in *E*.*coli* also interacts with PBP2 and/or RodA in the periplasm and thus couples cytoskeleton structure movement to peptidoglycan biosynthesis [50]. In previous work, we proposed that RodZ is essential in *B*. *subtilis* and that it is an important determinant of its rod cell shape [18]. We also observed direct interactions of RodZ with other cytoskeletal proteins including MreB, Mbl and MreBH and the morphogenetic protein MreD. The observation of these interactions suggests that RodZ is an integral part of a multi-protein complex that may help to spatially organize the proteins involved in peptidoglycan synthesis and turnover. In addition, RodZ influences cell division as demonstrated in the strains depleted for RodZ where as well as wider cells we observed smaller rounder cells, which in contrast to “minicells”, contained DNA [18]. We hypothesized that these cells arose from non-medial cell division implying that RodZ helps to block division at the poles in vegetatively growing cells [18,51].

In this work, we demonstrate the importance of RodZ in sporulation of *B*. *subtilis*. Our study began with the determination of the sporulation efficiency of strains in which RodZ production is perturbed. While in a strain expressing a truncated *rodZ* encoding its cytosolic domain, despite that the cells were wider and rounder, the sporulation efficiency is similar to that of wild type, a significant drop was detected in a strain depleted of RodZ by growth in the absence of the inducer IPTG. The magnitude of this effect might be underestimated since we were unable to prepare a null mutant of *rodZ* due to its essentiality. Taken together we conclude that the cytosolic domain of RodZ expressed at physiological levels is sufficient for both growth and sporulation.

In an effort to determine how RodZ affects sporulation, we first determined the level of Spo0A in cultures depleted of RodZ and found this to be similar to that in sporulating wild type strain (Fig 3C). Since Spo0A is the key regulator of initiation of sporulation, this result indicates that the cells have entered the sporulation pathway normally. Secondly, we followed the formation of asymmetric septa and the activation of σ^F^ in cultures depleted of RodZ. We observed a significant decrease in the proportion of cells exhibiting asymmetric septa, moreover we observed activation of σ^F^, albeit at low frequency, in undivided cells. The formation of an asymmetric septum is the first clear morphological event in sporulation and a prerequisite for subsequent activation of σ^F^, the first compartment-specific sigma factor [52]. The effect of RodZ depletion in some ways resembles that of *divIVA* deletion [53]. In the absence of DivIVA, asymmetric septation does not occur and σ^F^ is prematurely activated in a non-compartment-specific manner. This is due to disruption of SpoIIE function in the absence of DivIVA [53].

**Fig 6:**
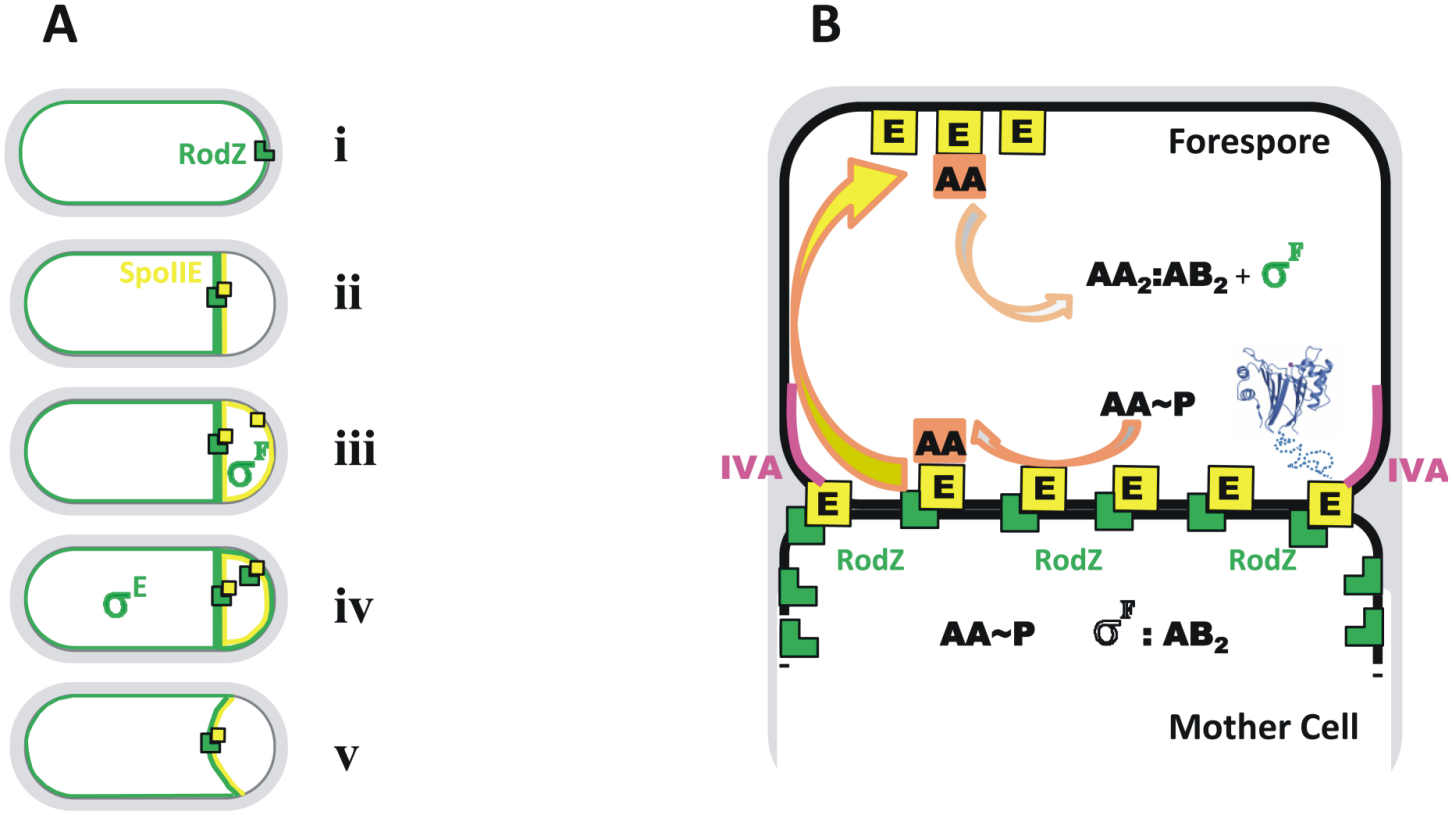
Model of RodZ function during sporulation. A) The distributions of RodZ and SpoIIE are indicated by the green and yellow lines respectively with representative molecules shown as green L shapes and yellow squares. (i) In addition to entire membrane localization, RodZ localizes at the poles (only one shown) of the cell during late stationary phase and later (ii) it relocalizes to the asymmetric septum site in a SpoIIE-dependent manner. Conversely, RodZ helps to localize and stabilize SpoIIE, likely as an integral part of a multi-protein complex. (iii) When the asymmetric septum is complete, SpoIIE is released from this site and redistributed throughout the forespore membrane. At this stage, RodZ is trapped in the septum and we propose that detachment of SpoIIE from RodZ and/or from the multi-protein complex might be connected with activation of σ^F^. (iv) Next, RodZ too is released from the septum but the purpose of this is not clear. Interestingly, recapture of SpoIIE and RodZ back to the septum seems to be simultaneous (v). As RodZ is linked to the peptidoglycan synthesizing machinery, it may take on a role in peptidoglycan thinning during engulfment. B) Compartment-specific activation of σ^F^ in the forespore. Upon completion of the asymmetric septum, in both the forespore and mother cell compartments, σ^F^ is inhibited by binding to the anti-σ^F^ factor, SpoIIAB (AB). The anti-sigma factor antagonist SpoIIAA (AA) is phosphorylated and inactive. The phosphatase SpoIIE (E) is shown at the asymmetric septum in a complex with RodZ. For a representative SpoIIE molecule on the right, the phosphatase domain is shown as a blue ribbon tracing. There is evidence to suggest that E dephosphorylates AA~P but that the dephosphorylated AA is trapped in complex with SpoIIE. Upon release of E from the septum into the forespore membrane (yellow arrow), E releases AA into the forespore cytoplasm (orange arrow) where it displaces σ^F^ from its complex with AB, allowing the former to combine with core RNA polymerase and initiate forespore-specific gene expression. This sets in train a signalling system that activates σ^E^ in the mother cell establishing the alternate programmes of gene expression that determine the different cell fates. The mechanism underlying the release of SpoIIE from the divisome/RodZ structure is not known. DivIVA which also localizes to the sporulation septum during stage II is marked in pink.

RodZ in *C*. *crescentus* co-localizes with FtsZ at the septum [48] while in *Chlamydiae* which have no FtsZ homologue, RodZ is also recruited to the division septum [54]. We found that *B*. *subtilis* RodZ besides localizing in helical patches, localizes at the cell poles and at the mid-cell site of septation in vegetatively growing cells [18]. During sporulation, RodZ is initially found at possible polar division sites, later RodZ accumulates at the polar septum and finally it is distributed around the membrane of forespores undergoing engulfment (Figs 2 and 6A).

To explore a possible relationship between RodZ and SpoIIE, we examined localization of RodZ and SpoIIE during sporulation. Comparison of RodZ and SpoIIE localization revealed that these two proteins co-localize at the asymmetric septum. Detailed analysis of their localization also showed that both proteins are transiently redistributed throughout the forespore membrane and as soon as engulfment is initiated, re-located to the septal membrane. While the function of SpoIIE associated with this release from the septum might be connected with activation of σ^F^ (Fig 6A and 6B), the purpose of RodZ release is not clear. It may be that RodZ helps to stabilize SpoIIE not only in complex with FtsZ, but also in some way in the membrane (Fig 3C). Recently it was demonstrated that SpoIIE localization and activity is restricted to the forespore through degradation by the ATP dependent protease, FtsH [55]. Thus, it is attractive to speculate that RodZ could protect SpoIIE from proteolysis by FtsH in the forespore and at the divisome. However, it is important to note that relative to SpoIIE release, RodZ release from the septum seems to be delayed. We observed that early in stage II, when the asymmetric septum is formed and SpoIIE has been released, RodZ is trapped in the septa in about one fifth of the cells. Interestingly, we did not observe the opposite situation where RodZ is released while SpoIIE remains in the septum. This suggests that the release of SpoIIE and RodZ are partially separable processes though recapture of SpoIIE and RodZ is simultaneous.

We can speculate that this dynamic RodZ localization at the polar septum reflects its further role in engulfment and its possible involvement in peptidoglycan remodelling (Fig 6A). If it is located in the outer and inner forespore membranes, RodZ may interact with the SpoIIDPM peptidoglycan remodelling complex [56–58] and/or the SpoIIIAH-SpoIIQ membrane migration / channel forming complex [59–61]. Indeed, the pattern of RodZ deposition and localization in the forespore membrane seems to correspond to the path taken by these engulfment complexes as they move around the forespore during membrane migration.

Further analysis of RodZ localization reveals, that RodZ does not localize properly in the absence of SpoIIE, but that it does localize correctly in the presence of the phosphatase activity deficient mutant SpoIIE64. This result indicates that RodZ might form a complex with SpoIIE and this is sufficient for proper localization and stabilization of RodZ during sporulation. In contrast, it was shown that DivIVA localizes to the polar septum independently of SpoIIE [53]. However, we cannot exclude a possible functional interplay of all three proteins. It is likely that a larger protein complex controls the asymmetric septum formation and σ^F^ activation.

Conversely, in a strain where RodZ is depleted by cell culture, and in which transcription from the *P*_*spoIIE*_ promoter is unaffected, the SpoIIE-Ypet fluorescence signal was too weak to allow us to determine whether SpoIIE localizes properly. Moreover the absence of a signal on Western blot indicates that the stability of SpoIIE-Ypet is affected in the absence of RodZ. It was reported recently that SpoIIE is degraded by the protease FtsH and is selectively stabilized in the forespore. This ensures that SpoIIE does not accumulate or become active prior to asymmetric division or in the mother cell following asymmetric division [55]. Thus it is tempting to speculate that RodZ plays an important role in this process. Interestingly, in a strain expressing a truncated *rodZ* encoding its cytosolic domain, SpoIIE localizes as in wild type cells even though these cells are wider and rounder. This is in agreement with the measured sporulation efficiency of this strain which, as mentioned above, is similar to that of wild type. In addition, the importance of the cytosolic part of RodZ is demonstrated by its interactions with the cytoskeletal proteins (MreB, MreBH, Mbl) [18] and as we show here, with the cytosolic part of SpoIIE.

Taken together, we suggest that formation of a RodZ-SpoIIE complex plays an important role in sporulation (Fig 6A and 6B). First, RodZ is likely involved in asymmetric septum formation and stabilization of SpoIIE in the septum. Second, release of SpoIIE from its complex with RodZ may be crucial for activation of σ^F^ specifically in the forespore. Third, due to its localization and connection to the peptidoglycan synthesizing machinery, it might be involved in peptidoglycan thinning during engulfment. The discovery of a role for the morphogenic protein RodZ in sporulation and its interaction with SpoIIE may help in the elucidation of the proposed function of SpoIIE in peptidoglycan remodelling. We can thus speculate that during engulfment, RodZ becomes an integral part of a multi-protein complex that orchestrates peptidoglycan remodelling. These results raise new questions concerning the possible role of cell shape-determining proteins in the process of spore formation.

## Supporting Information

S1 File Table A. Bacterial strains. Table B. Plasmids. Table C. Oligonucleotides.
(DOCX)
