# Supplementary material for: Morphogenic Protein RodZ Interacts with Sporulation Specific SpoIIE in Bacillus subtilis
Source: PLoS One. 2016 Jul 14;11(7):e0159076. doi: 10.1371/journal.pone.0159076 (PMC4945075; doi:10.1371/journal.pone.0159076)
Supplement: S1 File — Table A. Bacterial strains. Table B. Plasmids. Table C. Oligonucleotides. (DOCX) [file pone.0159076.s001.docx]

**S1 - Supporting information**

**Table A. Bacterial strains**

| **Strain** | **Genotype** | **Source/reference** |
| --- | --- | --- |
| *B. subtilis* |  |  |
| PY79 | Prototrophic derivative of *B. subtilis* 168 | ^*^ |
| MO1099 | *amy:: erm* | [35] |
| IB827 | *amyE::P_spoIIQ_-gfp* | P. Eichenberger |
| IB1563 | *amyE::P_spoIIQ_-gfp* *rodZ:: pMrodZ* | this work |
| PY180 | *spoIIE*::Tn*917*Ω*HU7* | [14] |
| IB1457 | PY79 *rodZ::* *pUKrodZ_91_* | [18] |
| IB1458 | PY79 *rodZ:: pMrodZ* | [18] |
| IB1459 | MO1099 *amyE:: P_xyl_-mgfprodZ spc* | [18] |
| IB1565 | *spoIIE*::Tn*917*Ω*HU7 amyE:: P_xyl_-mgfprodZ spc* | this work |
| PY507 | *spoIIE64 trpC2* | [44] |
| IB1566 | *spoIIE64 trpC2 amyE:: P_xyl_-mgfprodZ spc* | this work |
| IB1537 | *p_spoIIE_ spoIIE-ypet* | this work |
| IB1538 | *p_spoIIE_-spoIIEypet cat lacA::p_xyl_-cfp-rodZ erm* | this work |
| IB1567 | *p_spoIIE_-spoIIEypet rodZ::* *pUKrodZ_91_* | this work |
| IB1568 | *p_spoIIE_-spoIIEypet rodZ:: pMrodZ* | this work |
| IB1598 | PY79 *SPβ::P_spoIIE_-lacZ* | this work |
| IB1599 | PY79 *SPβ::P_spoIIE_-lacZ spo0A::kan* | this work |
| IB1600 | PY79 *SPβ::P_spoIIE_-lacZ rodZ:: pMrodZ* | this work |
| NB1418 | *lacA*::*cfp-rodZ erm* | this work |
| NB1641 | *lacA*::*cfp-rodZ erm amyE*::*spoIIE-3xflag spc spoIIE*::*kan* | this work |
| *E.coli* |  |  |
| MM294 | *F^−^ endA-1 hsdR-1, (rk^−^, mk) supE44 thi-1 recA1* | ^**^ |
| DH5α | *F^−^ Φ80lacZΔM15 Δ(lacZYA-argF) U169 recA1 endA1 hsdR17 (rK^–^, mK^+^) phoA supE44 λ– thi-1 gyrA96 relA1* | Invitrogen |
| BL21(DE3) | *hsdS gal (λcts857 indt Sam7 nin5 lacUV5-T7gene* | Novagen |
| BTH101 | *F^-^cya-99 araD139 galE15 galK16 rpsL1(Str ^r^)hsdR2 mcrA1 mcrB1* | [36] |

**Table B. Plasmids**

| **Plasmid** | **Description** | **Reference** |
| --- | --- | --- |
| pSGrodZ | *bla amyE spc P_xyl_-mgfprodZ amyE* | [18] |
| pAX01 | *bla lacA erm P_xyl_ lacA* | [34] |
| pAXrodZ | *bla lacA erm P_xyl_-cfprodZ lacA* | this work |
| pSG1151 | *cla cat gfpmut1* | [33] |
| pSG1151IIE-Ypet | *bla cat P_IIE_-spoIIE(724-827aa)-ypet* | this work |
| pDG1728 | *bla amyE spc amyE* | [35] |
| pDGIIE-3xFlag | *bla amyE spc P_IIE_-spoIIE-3XFLAG amyE* | this work |
| pETDuet-1 | expression vector used for proteins co-expression, *bla lacI T7 promoter* | Novagen |
| pRSFDuet-1 | expression vector used for proteins co-expression, *bla lacI T7 promoter* | Novagen |
| pETrodZ | *bla lacI P_T7_cytrodZ* | [18] |
| pETspoIIE-S | *bla lacI P_T7_cytspoIIE-S* | this work |
| pETrodZspoIIE-S | *bla lacI P_T7_cytrodZcytspoIIE-S* | this work |
| pETspoIIE | *bla lacI P_T7_cytspoIIE* | this work |
| pKT25 | enable fusion to C-terminal end of T25 fragment of adenylate cyclase | [36] |
| pKNT25 | enable fusion to N-terminal end of T25 fragment of adenylate cyclase | [36] |
| pUT18 | enable fusion to N-terminal end of T18 fragment of adenylate cyclase | [36] |
| pUTC18 | enable fusion to C-terminal end of T18 fragment of adenylate cyclase | [36] |
| pKTrodZ | *P_lac_-T25-rodZ kan* | [18] |
| pKNTrodZ | *P_lac_-rodZ-T25 kan* | [18] |
| pUTrodZ | *P_lac_-rodZ-T18 bla* | [18] |
| pUTCrodZ | *P_lac_-T18-rodZ bla* | [18] |
| pKTcytrodZ | *P_lac_-T25-cytrodZ kan* | [18] |
| pKNTcytrodZ | *P_lac_-cytrodZ-T25 kan* | [18] |
| pUTcytrodZ | *P_lac_-cytrodZ-T18 bla* | [18] |
| pUTCcytrodZ | *P_lac_-T18-cytrodZ bla* | [18] |
| pKTIIE | *P_lac_-T25-spoIIE bla* | this work |
| pKNTIIE | *P_lac_-spoIIE-T25 bla* | this work |
| pUTIIE | *P_lac_-spoIIE-T18 bla* | this work |
| pUTCIIE | *P_lac_-T18-spoIIE bla* | this work |
| pKTnIIE | *P_lac_-T25-spoIIE domain I bla* | this work |
| pKNTnIIE | *P_lac_- spoIIE domain I-T25 bla* | this work |
| pUTCnIIE | *P_lac_-T18- spoIIE domain I bla* | this work |
| pUTnIIE | *P_lac_- spoIIE domain I-T18 bla* | this work |
| pKTnctIIE | *P_lac_-T25- spoIIE domain I,II bla* | this work |
| pKNTnctIIE | *P_lac_-spoIIE domain I,II-T25 bla* | this work |
| pUTCnctIIE | *P_lac_-T18-spoIIE domain I,II bla* | this work |
| pUTnctIIE | *P_lac_-spoIIE domain I,II T18 bla* | this work |
| pKTctIIE | *P_lac_-T25-spoIIE domain II bla* | this work |
| pKNTctIIE | *P_lac_-spoIIE domain II-T25 bla* | this work |
| pUTCctIIE | *P_lac_-T18-spoIIE domain II bla* | this work |
| pUTctIIE | *P_lac_-spoIIE domain II-T18 bla* | this work |

**Table C. Oligonucleotides**

| **Primer** | **Sequence 5′- 3**′ |
| --- | --- |
| cytspoIIESX | GATGATGATCTCGAGATGATTCCGGGAACTGTC |
| cytspoIIEEX | GATGATGATCTCGAGTGAAATTTCTTGTTTGTTTTGA |
| cytspoIIESB | GATGATGATGGATCCGATGATTCCGGGAACTGTC |
| cytspoIIEEB | GATGATGATGGATCCGGTTATGAAATTTCTTGTTTGTTTTGA |
| spoIIEXba  forw2 | GATGATGATTCTAGACATGGAAAAAGCAGAAAGAAGAGTGAAC |
| spoIIEKpn  Rev | GATGATGATGGTACCGATGAAATTTCTTGTTTGTTTTGAAAGATTGCCGGAAC |
| IIENEndpnR | ATCATCATCGGTACCCTCGCCACTTTCCTCGTAATAGATTG |
| IIECentrEnd  KpnR5 | ATCATCATCGGTACCGAGACAAGGCCGCCGCCCTTC |
| IIEcytKpnF3 | ATCATCATCGGTACCCAATACGCTAGAAAAATCCGTGATG |
| IIECpxNcoF2 | GATGTCTTTCCATGGCAATCAGTGACGGAATGGGCAATG |
| IIECpxKpnR | GATGATGATGGTACCGTATGAAATTTCTTGTTTGTTTTGAAAGATTG |
| YpetKpnFor2 | ATCATCATCGGTACCATGTCTAAAGGTGAAGAATTATTCACTGGTGTTGTC |
| YpetPstRev2 | ATCATCATCCTGCAG TTATTTGTACAATTCATTCATACCCTC |
| CFP-pAX01f | GATATCTAAAAATCAAAGGGGGAAATGGGATCCACATAAGGAGGAACTACTATGGTTTC |
| CFPr | TGTAATGCCCGCAGCTG |
| CFP-RodZf | CAGCTGCGGGCATTACAATGTCATTGGATGATCTCCAAGC |
| RodZ-AX01r | GAAGAGTGCGGCCGCCCGCGGGATTGGGATTAATGCGATTCTAGC |

The underlined parts refer to restriction sites.

* Youngman P, Perkins JB, Losick R. Construction of a cloning site near one end of Tn917 into which foreign DNA may be inserted without affecting transposition in Bacillus subtilis or expression of the transposon-borne *erm* gene. Plasmid. 1984;12: 1-9. doi: 10.1016/0147-619x(84)90061-1.

** Backman K, Ptashne M, Gilbert AW. Construction of plasmids carrying the cI gene of bacteriophage lambda. Proc Natl Acad Sci USA. 1976;73: 4174-4178. . doi: 10.1073/pnas.73.11.4174.
